# ASO Author Reflections: Peritoneal Metastases from Endometrial Cancer

**DOI:** 10.1245/s10434-018-6819-5

**Published:** 2018-10-04

**Authors:** Paolo Sammartino, Tommaso Cornali

**Affiliations:** 1 Department of Surgery “Pietro Valdoni”, Sapienza University of Rome, Rome, Italy; 2 Department of Surgery “Pietro Valdoni”, Azienda Policlinico Umberto, Rome, Italy

## Past

Although withdrawn as a criterion for staging endometrial cancer (EC), the American Joint Committee on Cancer (AJCC) recommends the detection of neoplastic cells in peritoneal washings as level I evidence. Intraluminal tumor cells (ILTCs) in the fallopian tubes (a histological marker of transtubal spread), detected in 10–20% of ECs, were associated with aggressive tumors, positive peritoneal cytology, and decreased survival in serous EC and early-stage, low-risk EC.^1^,^2^ Some evidence suggests the need for early bilateral tubal ligation before surgical maneuvers, whereas conflicting data relates to the possible use of a uterine manipulator and removing the uterus through the vagina, all procedures theoretically correlated with possible tumor cell spillage into the peritoneum. Data from an observational retrospective cohort analysis from the National Cancer Database including women with a known cytology status and AJCC stage IA–II EC showed that positive peritoneal washing cytology indicated decreased overall survival and the need for adjuvant chemotherapy to improve outcome.^3^

## Present

Collective reviews reporting a series of patients who underwent cytoreductive surgery (CRS) combined with hyperthermic intraperitoneal chemotherapy (HIPEC) for peritoneal metastases from various primary diseases included some with EC, but they failed to analyze their features in detail. The few patients with EC who had peritoneal metastatic spread during the natural history of the disease, and who could be referred to tertiary centers specialized in treating peritoneal surface malignancies (PSM) with CRS plus HIPEC, make it impossible to assess the value of this therapeutic strategy in randomized trials. Despite being retrospective, our study, contributes previously unavailable, detailed evidence-based information on the treatment of peritoneal metastases from EC with CRS plus HIPEC. Especially when CRS leaves no residual disease, this combined treatment, given in experienced centers to properly selected cases, achieves outcome rates approaching those for the most frequent indications (colorectal, ovarian cancer).^4^

## Future

According to a French Multicenter Database including data for classifying recurrent EC,^5^ metachronous peritoneal spread had the worst outcome compared with other recurrence pathways, with a significantly lower median survival than we obtained in our patients who underwent CRS plus HIPEC (8 vs. 33 months). Given that most peritoneal metastases in patients with EC develop after primary surgery, we need to intensify efforts to improve risk assessment by integrating molecular and clinicopathological factors (peritoneal cytology, histology subtype, grading, lymphovascular space invasion, p53 expression, and microsatellite instability). Unfortunately, the only diagnostic tool that can reliably predict the risk of peritoneal spread in an intraoperative setting in patients with EC is peritoneal cytology. After reflecting on essential steps in improving outcomes, we suggest that peritoneal washings should be collected when surgical procedures begin and end. If peritoneal cytology yields positive findings, the patient should undergo omentectomy and peritoneal port system placement to integrate systemic adjuvant chemotherapy with intraperitoneal chemotherapy.
